# Adaptation of health care for migrants: whose responsibility?

**DOI:** 10.1186/1472-6963-14-294

**Published:** 2014-07-08

**Authors:** Marie Dauvrin, Vincent Lorant

**Affiliations:** 1 Institute of Health and Society IRSS, Université catholique de Louvain, Clos Chapelle aux Champs 30 boîte 1.30.15, Brussels 1200, Belgium; 2 Fonds de la Recherche Scientifique-FNRS, rue d’Egmont 5, Bruxelles 1000, Belgium

**Keywords:** Responsibility, Cultural competence, Health professionals, Factor analysis, Multilevel model, Negotiation of values, Communication, Health beliefs, Migrants

## Abstract

**Background:**

In a context of increasing ethnic diversity, culturally competent strategies have been recommended to improve care quality and access to health care for ethnic minorities and migrants; their implementation by health professionals, however, has remained patchy. Most programs of cultural competence assume that health professionals accept that they have a responsibility to adapt to migrants, but this assumption has often remained at the level of theory. In this paper, we surveyed health professionals’ views on their responsibility to adapt.

**Methods:**

Five hundred-and-sixty-nine health professionals from twenty-four inpatient and outpatient health services were selected according to their geographic location. All health care professionals were requested to complete a questionnaire about who should adapt to ethnic diversity: health professionals or patients. After a factorial analysis to identify the underlying responsibility dimensions, we performed a multilevel regression model in order to investigate individual and service covariates of responsibility attribution.

**Results:**

Three dimensions emerged from the factor analysis: responsibility for the adaptation of communication, responsibility for the adaptation to the negotiation of values, and responsibility for the adaptation to health beliefs. Our results showed that the sense of responsibility for the adaptation of health care depended on the nature of the adaptation required: when the adaptation directly concerned communication with the patient, health professionals declared that they should be the ones to adapt; in relation to cultural preferences, however, the responsibility felt on the patient’s shoulders. Most respondents were unclear in relation to adaptation to health beliefs. Regression indicated that being Belgian, not being a physician, and working in a primary-care service were associated with placing the burden of responsibility on the patient.

**Conclusions:**

Health care professionals do not consider it to be their responsibility to adapt to ethnic diversity. If health professionals do not feel a responsibility to adapt, they are less likely to be involved in culturally competent health care.

## Background

The increasing ethnic diversity of populations in Europe has transformed the delivery of health care and the habits of health professionals [1]. Health professionals are required to adapt to specific demands by migrant patients in order to lower linguistic or cultural barriers that prevent these groups from accessing adequate care [2,3]. This adaptation requirement is reinforced by international and national recommendations. In an increasingly multicultural Europe, health systems, health services, and health professionals are under pressure to become “*migrant-friendly*” [4-7]. Indeed, the Committee of Ministers of the Council of Europe [5] recommended that “*the governments of the member states promote the involvement and participation of all parties concerned (researchers, policy makers, local health authorities, health professionals, representatives of ethnic minority groups and non-governmental organisations) in the planning, implementation and monitoring of health policies for multicultural populations”*. This involves a wide range of strategies: health policies, the deployment of interpreters and intercultural mediators in health facilities, the development of culturally-specific health services or ethnically sensitive health promotion campaigns, etc. [8-10] (see Table 1). All these strategies concern the individual, institutional, and political levels and interact in order to reduce the gap between migrant and non-migrant populations [11-13].

**Table 1:** Examples of culturally competent interventions

**Level**	**Content of the level**	**Examples of interventions**
A. Health care policy	*Cultural competence is operationalised chiefly through health policies and action plans for diversity in health care, but also through the labour market and in education [12, 13].*	•Standards of quality that take into account ethnic diversity, such as the CLAS standards in the United States, the NHS Checking for Change programme, and the Swiss Federal Strategy for health of migrants
		•Accreditation and licensing of health care professionals in cultural competence
		•Regulations and specific funding for culturally competent interventions such as intercultural mediation in Belgium
B. Health care organisation	*Adequacy of health care facilities in relation to the needs of migrant and minority patients, including both structural aspects of hospitals and training for health professionals [42].*	•Provision of religious facilities in health services
		•Recruitment policies
		•Hiring of interpreters or intercultural mediators
		•Culturally specific health services
		•Specific adaptations within the health services: providing meals that respect various religious practices, collaboration with traditional healers and religious leaders, etc.
C. Individual health care professional	*Cultural competence is operationalised through the development of knowledge, attitudes, and skills that aim to increase the quality of health care delivered to migrant patients within a culturally sensitive perspective [32].*	•Training in cultural competence
		•Ethnically sensitive educational material
		•Empowerment of patients

However, despite efforts to implement and broaden these strategies, disparities still persist in the quality of care and access to health care between ethnic minorities and the rest of the population [14-17]. Although the organisation of health services and health systems varies from one country to another, migrants and ethnic minorities still have lower levels of access to health promotion facilities and health prevention [18,19]. They also experience worse health outcomes in acute [20] and chronic conditions, such as type 2 diabetes mellitus [21] and asthma [22]. This leads to temporary or permanent complications, increasing the burden of diseases among these vulnerable groups: e.g. higher rates of amputations due to inadequate management of type 2 diabetes mellitus have been reported among ethnic minorities [23].

One way to reduce these ethnic inequities is to make health professionals modify their attitudes and become culturally competent [8,9,12]. Culturally competent health care is defined “*as one that acknowledges and incorporates—at all levels—the importance of culture, assessment of cross-cultural relations, vigilance toward the dynamics that result from cultural differences, expansion of cultural knowledge, and adaptation of services to meet culturally unique needs”* (from Betancourt *et al*. 2003, p 294 [9]). Becoming culturally competent involves a broad spectrum of interventions and strategies, requiring various degrees of adaptation, from marginal changes to radical ones [8,9,24-27] (see Table 1). It also requires health professionals to acknowledge their responsibility to provide solutions. Awareness of ethnic diversity and being aware that a solution is needed are the first two steps in most models of cultural competence [28-31]. Being aware of ethnic diversity assumes that health professionals acknowledge that their patients are heterogeneous and that their cultural background is likely to influence their perceptions and health behaviours. Moreover, health professionals are also required to identify their own cultural and professional background and the consequences of this identity for their practice. In that context, the second step, “*being aware that a solution is needed*”, involves the health professionals acknowledging that biases, prejudices, or non-adapted attitudes may occur when encountering a migrant patient. Most programmes aimed at developing cultural competence focus on health professionals or the health system as the actors responsible for adaptation [28,29,31,32]. These programmes assume that once health professionals are aware of the need for cultural competence, they will also feel a responsibility to adapt their behaviour. This assumption has often remained at the level of theory and it is still unclear whether health professionals feel responsible for the entire process of adaptation to ethnic diversity or whether they feel that this responsibility is shared with patients. In the patient-health professional encounter, the health professional is the person who is most knowledgeable about clinical matters and technical interventions, because the health professional has been trained in these fields. However, when it comes to the attitudes and behaviour of health professionals in terms of empathy, kindness, a sense of responsibility, and patient-centeredness, it is generally assumed that those attitudes and kinds of behaviour are mostly intrinsic to the persons concerned and are not always attitudes learned during training [33]. Although progress has been made in delivering patient-centred medical education, in practice the biomedical model still dominates the organisation and delivery of care. In Belgium, the training curricula of physicians and, to a lesser extent, of nurses is mostly focused on specialty medicine, with a low emphasis on family medicine, which is traditionally more patient-centred [34]. In this study, we set out to assess whether health professionals feel responsible for adapting health services to cope with a multicultural society. We carried out a survey of health professionals in order to describe who feels a responsibility to adapt in health care situations that involve cultural diversity.

## Methods

### Design and participants

This study is part of a larger research project – COMETH (Competence in Ethnicity and Health) – that aims to evaluate and understand the cultural competence of health professionals in Belgium. We used a purposive cluster sampling model where the cluster was a health service. First, we selected three geographical zones, according to the number of foreign residents, in Brussels and Wallonia (Belgium) [35]. Foreign residents were defined according to the definition used in the national statistics of the Belgian Ministry of Economy: “*a foreign resident is a non-Belgian resident with a legal permit of residence in Belgium*” [35]^1^. This definition included recognised refugees but excludes registered asylum-seekers and irregular migrants (e.g. migrants with an overstayed visa or migrants who do not fulfil the requirements to apply for asylum).

The zones were categorised in low exposure to immigration (proportion of foreign residents of 6.6% and below), medium exposure (proportion of foreign residents between 6.7% and 18.7%), and high exposure (proportion of foreign residents of 18.8% and higher) [35]. In 2011, at the national level, the resident foreign population represented 10.2% of the entire population [35]. Sixty-seven per cent of foreign residents are from the EU-27; Moroccans, Turks, and Congolese are the three main foreign nationalities outside the EU-27 [36].

In each geographical zone, we selected the largest hospital and the largest primary care service, according to the annual number of registered patients. In each hospital, we recruited at least four services: a chronic patient-centred care service, an acute patient-centred care service, a chronic non-patient-centred care service, and an acute non-patient-centred care service [37-39].

Two hospitals and one primary care service declined participation in the project. Two hospitals did not have a communicable diseases unit. In one hospital, the psychiatric unit declined to participate in the project due to an internal reorganisation at the time of the study. We ended up with 5 primary care services and 4 hospitals, including 19 inpatient health services. The 19 inpatient health services were: 4 geriatric units, 4 intensive care units, 4 oncology units, 3 psychiatric units, 2 communicable diseases units, 1 palliative care unit, and 1 endocrinology unit.

All health professionals and volunteers working in the service were invited to participate in the research. Preliminary consent was obtained from the management of each institution. We then met the physician and the nurse in charge of each selected service to obtain further consent. When this consent was delivered, we met the whole team to present the project and asked them for their consent. Participants were free to withdraw their consent during the survey process.

Data were collected with a self-administered questionnaire from June 2010 to June 2012. Participants had one month to complete the questionnaire. The introductory statement of the questionnaire recalled the objectives of the research and defined what we meant by “*migrant*”. We defined a migrant as “*first-, second-, and third- generation migrants, undocumented migrants, asylum seekers, refugees, ethnic minorities, cultural minorities, returned nationals, religious minorities…but excluding other types of minorities such as sexual minorities*” [40]. Reminders were sent to the head nurse and the doctor-in-chief of each service each week during the one-month data collection period.

This study was approved by the Hospital University Ethics Committee of the Université catholique de Louvain and by Belgium’s national Privacy Commission (Ordinary Declaration No. 1304326281174). As our database includes nominations of the respondents, access to data requires the authorisation of the Privacy Commission.

### Measures

Our dependent variable was the relative sense of responsibility for adaptation of health care. We used and adapted the scale of relative responsibility developed and validated by Hudelson and colleagues in Switzerland [41]. The scale of relative responsibility contains 5 semantic differential items (Table 2): adaptation to the values of the host country, the provision of interpreters, patient’s wishes to be treated by a male or a female doctor, the provision of written information in a language acceptable to the patient, and adaptation to health beliefs. Each item ranges from 1 to 7, 1 meaning full agreement that adaptation is the responsibility of the health professional and 7 meaning full agreement that adaptation is the responsibility of the patient. The respondents were asked to take a position on whether the adaptation was the responsibility of the patient or the responsibility of the health professional.

**Table 2:** **Scale of relative responsibility towards adaptation of health care for migrants, adapted from Hudelson *et al. *** 2010*[41]

*Instructions: In the following propositions, you may choose between two possible attitudes when encountering migrant patients in your health service. Please, choose the statement corresponding to your opinion (one answer by proposition).*								
**A.When immigrants' values and habits differ from those of the host country**								
Host country institutions should adapt to the immigrants ‘values and habits	1	2	3	4	5	6	7	Migrants should adapt to the values and habits of the host country
**B.When the patient does not speak the language of the host country**								
The health professional should always provide a professional interpreter	1	2	3	4	5	6	7	It is the patient’s responsibility to find an interpreter
**C.When the patient expresses the wish to be treated by a male of a female health professional**								
Hospitals should allow patients who request it to choose their health professional’s sex	1	2	3	4	5	6	7	Patients should accept to be treated by the health professional provided by the hospital, regardless of their sex
**D.When the patient cannot read the language of the host country**								
Hospitals should provide written information in the patient’s language	1	2	3	4	5	6	7	The patient should arrange to translate written information provided by the hospital
**E.When the patient's health beliefs contradict knowledge of the health professionals**								
The health professional should adapt to the patient’s beliefs regarding the disease and the treatment	1	2	3	4	5	6	7	The patient should trust the explanations and recommendations of the health professional

A score below 4 implies that the responsibility is placed on the health professionals or the health institutions while a score over 4 implies that the responsibility is placed on the patients. A score of 4 corresponds to an intermediate attitude in which the responsibility is shared by the patient and the health professional.

The psychometric properties of the scale and the similarities between the Belgian and Swiss contexts were decisive in its adoption as an instrument for data collection. The Cronbach’s α coefficient of the scale was 0.65. The test-retest intra-class correlation coefficient was 0.83 (CI95% 0.78-0.88). We obtained the consent of Patricia Hudelson to use her scale.

Our independent variables included objective and subjective exposure to migrant and ethnic minority groups, individual sociodemographic factors, and service-level factors. Objective exposure was measured by the localisation of the service in geographical zones with low exposure to immigration, medium exposure, or high exposure. Subjective exposure was measured by the frequency of exposure to intercultural situations as experienced by the health professionals. We developed a specific scale, adapted to the Belgian context. This scale was based on previous surveys in Belgium and Europe [42-44]. We performed specific factor analysis for the scale of subjective exposure to obtain final variables for analysis. The development of the scale is beyond the scope of this paper and is not presented here.

The individual sociodemographic factors were gender (male/female), age (mean age and by categories), nationality (Belgian, EU 27-European (from a member state of the European Union^2^), and non-European), languages spoken (national Belgian languages, i.e. French and Dutch, other European languages, and non-European languages), country of birth (Belgium, EU 27-European, and non-European), country of childhood (Belgium, EU 27-European, and non-European), and languages spoken with parents (national Belgian languages, i.e. French and Dutch, other European languages, and non-European languages). Professional characteristics were: professional qualification (nurses or care assistants, physicians, allied health professionals, administrative and social workers – including social assistants, volunteers, and faith assistants), working hours (full-time versus part-time), years of experience (by categories), additional training (additional qualification for nurses or physicians), culturally competent education (at school, at the hospital, or by personal choice), professional experience abroad (in an OECD country or in a non-OECD country), and additional functions in the service: head of service, clinical referent, non-clinical referent (e.g. informatics), student supervisor, others.

Service-level factors were the paradigm of care (patient-centred care versus non-patient-centred care), the length of stay (acute unit versus chronic unit), the type of service (intensive care, geriatrics, psychiatry, oncology, including the palliative care and endocrinology units, communicable diseases units, and primary care services), and the institution.

### Analysis

The analysis unfolded in three stages. First, we computed descriptive statistics. Second, we did a factorial analysis to analyse the correlations across the relative responsibility items. We also used factor analysis to convert the items into a continuous score of relative responsibility. After the factor analysis, we tested the normality of the distribution of the factors and decided to conserve the loading factors in the further analysis. Finally, we regressed the dependent variables resulting from the factor analysis on independent variables at the individual and institutional levels. We included all the variables in a regression model with a stepwise procedure to select the significant variables (at alpha = 10%) in the final analysis. Based on the results of the regression model, we fitted a mixed regression model to capture within-service and within-institution clustering. Sixteen observations had missing values and were not included in the regression models. Analyses were made with the SAS 9.2 software.

## Results

We contacted 872 members of the staff of the 24 services and we received 569 replies, yielding a participation rate of 65%. We compared participation rates across professional groups and found no statistical differences, particularly between nurses (62%) and physicians (53%). Most of the respondents were nurses or care assistants (59.7%, see Table 3), administrative and social workers (16%), physicians (12.3%), and allied health professionals (12%). Our sample reflected the distribution of health professionals in Belgian health services [45,46]. Most of the respondents were Belgian. Sixteen per cent of the participants had a high level of exposure to migrant patients.

**Table 3:** Sociodemographic characteristics of the health professionals of the COMETH study conducted in Belgium in 2010–2012 (n=569)

	**Number**	**(%)**
**Profession**		
Nurses and care assistants	340	59.7
Administrative and social staff	91	16.0
Physicians and medical assistants	70	12.3
Allied health professionals	68	12.0
**Sex**		
Women	448	78.7
Men	121	21.3
**Age groups (years)**		
20-29 y	144	25.3
30-39 y	154	27.1
40-49 y	140	24.6
50-59 y	104	18.3
60y and more	27	4.7
**Objective exposition to migration**		
Low	250	43.9
Medium	228	40.1
High	91	16.0
**Subjective exposition to migration (n = 563)**		
Very low	114	20.2
Low	173	30.7
Medium	187	33.2
High	89	15.8

Table 4 displays the distribution of the relative responsibility for adaptation in health care according to the health professionals.

**Table 4:** Reported attitudes of health professionals about the relative responsibility towards adaptation of health care in the COMETH study in Belgium in 2010–2012 (n = 569)

	**The responsibility is on the health professionals**	**The responsibility is on both health professionals and patients**	**The responsibility is on the patients**	**Total**
When immigrants' values and habits differ from those of the host country (%)	20.7	18.2	61.0	100.0
When the patient does not speak the language of the host country (%)	65.3	19.7	15.0	100.0
When the patient expresses the wish to be treated by a male of a female doctor (%)	34.3	10.4	55.3	100.0
When the patient cannot read the language of the host country (%)	49.0	19.5	31.5	100.0
When the patient's health beliefs contradict medical knowledge (%)	36.8	27.0	36.1	100.0

We found that different features were associated with different views on responsibility. The provision of interpreters and written information were reported as being the responsibility of health professionals, while patients were expected to adapt to the gender of health professionals and to the values of the host country. Indeed, more than half of the respondents (55.3%) reported that patients had to adapt to the gender of health care professionals. We found that most respondents were unclear about adaptation to health beliefs: a third (36.8%) thought that the responsibility was the health care professional’s, while 36% thought the responsibility was the patient’s.

**Figure 1:**
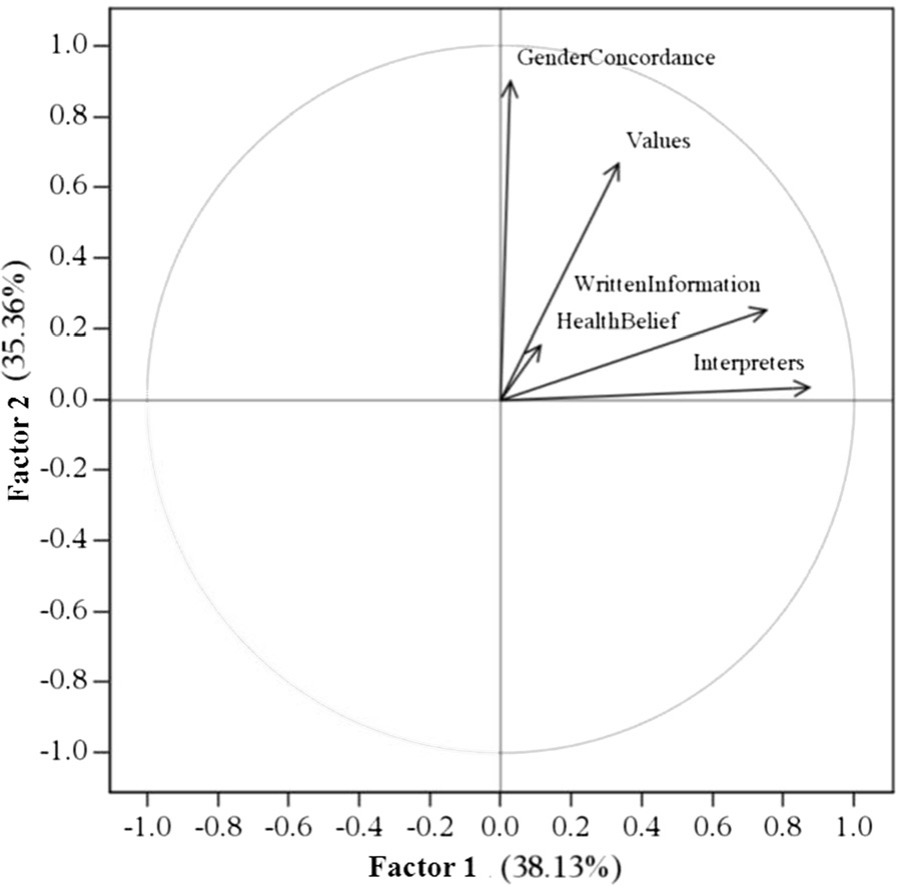
**The horizontal axis (Factor 1) is influenced by the items “Written Information” and “Interpreters”.** The horizontal axis is resumed by “*Responsibility for adaptation to instrumental communication*”. The vertical axis (Factor 2) is influenced by the items “Gender Concordance” and “Values”. The vertical axis is resumed by “*Responsibility for adaptation to the negotiation of values*”.

**Figure 2:**
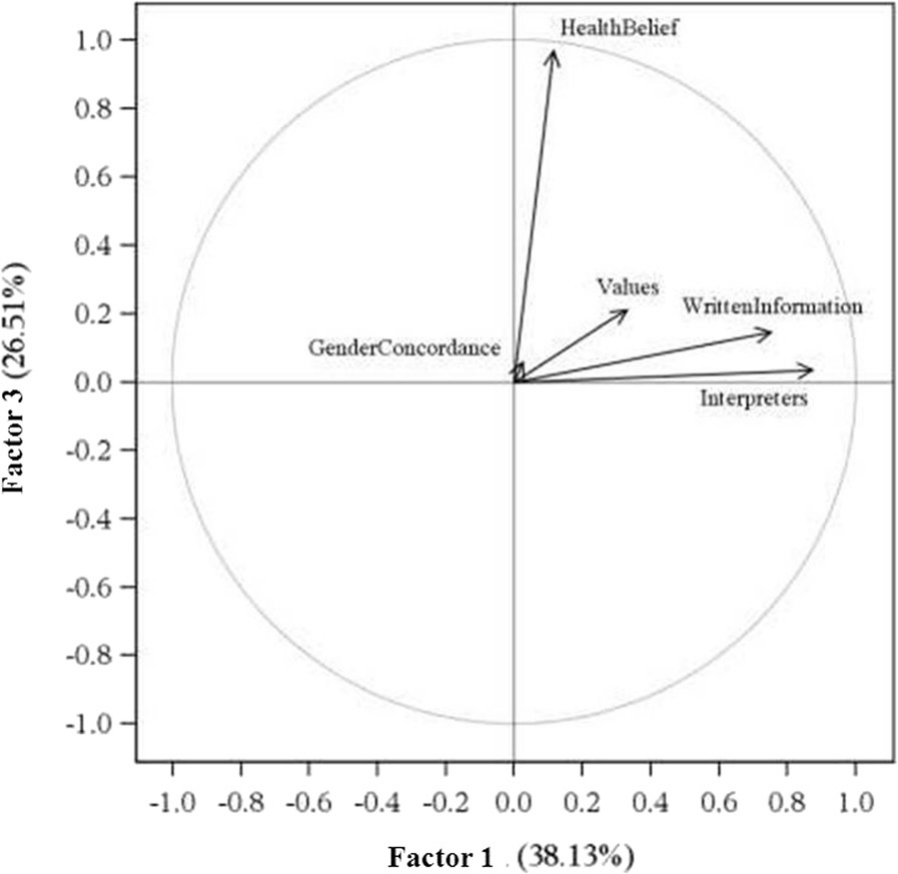
**The horizontal axis (Factor 1) is influenced by the items “Written Information” and “Interpreters”.** The horizontal axis is resumed by “*Responsibility for adaptation to instrumental communication*”. The vertical axis (Factor 3) is influenced by the item “Health Belief”. The vertical axis is resumed by “*Responsibility for adaptation to health beliefs*”.

**Figure 3:**
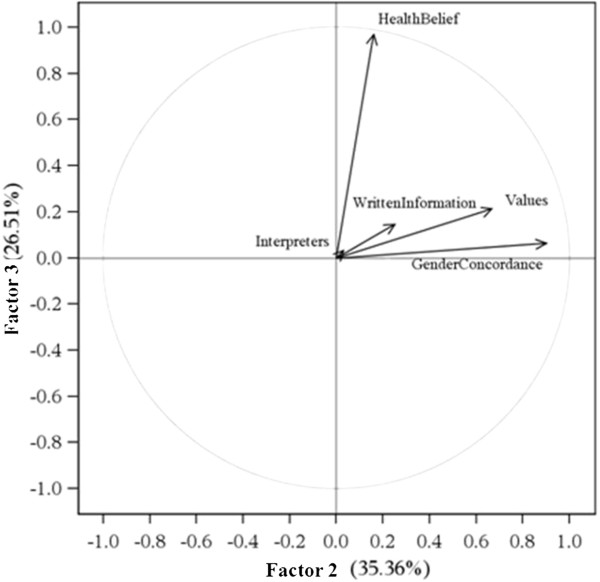
**The horizontal axis (Factor 2) is influenced by the items “Gender Concordance” and “Values”.** The horizontal axis is resumed by “Responsibility for adaptation to the negotiation of values”. The vertical axis (Factor 3) is influenced by the item “Health Belief”. The vertical axis is resumed by “Responsibility for adaptation to health beliefs”.

We compared the attribution of responsibility according to the professional background of the respondents. We did find small but non-significant differences between the various professions in our sample. Physicians declared themselves more responsible for providing interpreters (mean score 2.8 ± 1.39) and written information (mean score 3.17 ± 1.44) to migrant patients than other health professions (mean score for paramedics 3.12 ± 1.55; mean score for administrative staff 3.44 ± 1.82; mean score for nurses 2.82 ± 1.57). Nurses and care assistants expected more often that the patients should adapt to the gender of health professionals (mean score 4.69 ± 2.01) than other health professionals (mean score for paramedics 4.44 ± 2.03; mean score for administrative staff 4.39 ± 2.15; mean score for physicians 4.39 ± 2.07).We also compared the attribution of responsibility between the formal heads of services and the rest of the health professionals. We did find small but non-significant differences between the two groups. Heads of services tend to consider health professionals more responsible for adapting health care for migrants than the rest of the health professionals.Based on the scree plot after Varimax rotation, we retained three factors (see Figure 1, Figure 2 and Figure 3). Factor 1 was correlated with item 2, “providing interpreters” (0.88), and item 4, “providing written information” (0.76). Factor 2 was correlated with item 1, “values of the host country” (0.67) and item 3, “gender concordance” (0.90). Factor 3 was correlated with item 5, “health beliefs” (0.97).

We interpreted factor 1 as “*Responsibility for adaptation to instrumental communication*” as it comprised two items related to communication (the provision of interpreters and the provision of written information). We interpreted factor 2 as “*Responsibility for adaptation to the negotiation of values*”, because it comprised items related to the values of the host country and gender concordance. Factor 3, interpreted as “*Responsibility for adaptation to health beliefs*”, comprised item 5: health beliefs.

Each factor resulting from the factor analysis was integrated into a regression model in order to identify the predictive covariates of responsibility (Table 5). Overall, no independent variable was common to the three responsibility factors, although some independent variables were common to two responsibility factors. Each score ranged from -2 to 2. A score below 0 implied that the responsibility was attributed to the health professional. A score over 0 implied that the responsibility was attributed to the patients.

**Table 5:** Who is responsible for adapting health care to diversity, according to the health professionals in the COMETH study in Belgium in 2010–2012 : Covariates of responsibility, Beta and 95% Confidence Intervals from the mixed regression model (n = 569)

	**Score of responsibility according to the factor analysis**		
	*Score of responsibility for adaptation to the instrumental communication* [-2; +2]	*Score of responsibility for adaptation to the negotiation of values* [-2; +2]	*Score of responsibility for adaptation to the health beliefs* [-2; +2]
**Covariates of responsibility**	Beta [95% CI]	Beta [95% CI]	Beta [95% CI]
*Professional background*			
Physicians	-0.31 [-0.55; -0.10]	-	-0.49 [-0.70; -0.29]
Other professions^±^	0.00	-	0.00
*Nationality*			
Belgian	0.46 [0.19; 0.71]	0.32 [0.06; 0.57]	-
Non Belgian^±^	0.00	0.00	-
*Type of health service*			
Intensive care unit	-	-	0.34 [0.19; 0.49]
Not an intensive care unit^±^	-	-	0.00
Primary care service	0.52 [0.25; 0.79]	-0.52 [-0.78; -0.25]	-
Not a primary care service^±^	0.00	0.00	-
**Covariates estimates**			
Random intercept^┼^	0.04	0.04	-
	p = 0.073	p = 0.08	
Residual	0.91	0.93	0.94
	p < 0.0001	p < 0.0001	p < 0.0001

^±^Indicates the reference category- Indicates non-significant results ^┼^random intercept = service.

A score below 0 implied that the responsibility was attributed to the health professionals.

A score over 0 implied that the responsibility was attributed to the patients.

Compared to non-Belgians, Belgians placed more responsibility on patients than on health professionals or the health services in relation to the negotiation of values and instrumental communication. Compared to the other professions, physicians placed more responsibility on health professionals than on patients in relation to instrumental communication and adaptation to health beliefs. When compared to health professionals working in another health service, health professionals working in primary care placed more responsibility on the patients than on health professionals in relation to communication. When compared to health professionals working in another health service, health professionals working in primary care service placed more responsibility on health professionals than on patients in relation to the negotiation of values. When compared to health professionals working in another health service, health professionals working in an intensive care unit placed more responsibility on patients than on health professionals in relation to adaptation to health beliefs.

## Discussion

We surveyed health professionals in 24 health services to find out who was considered to be responsible for adapting health services to a multicultural society. The results imply that attitudes to responsibility for the adaptation of health care depend on the nature of the adaptation required: when the adaptation directly concerned communication with the patient, health professionals declared they are the ones to adapt, but for cultural preferences the responsibility seems to fall on patients’ shoulders. For responsibility regarding communication, primary care services placed more responsibility on patients than on health professionals when compared to non-primary care services; but for the negotiation of values it was the other way round.

How can we explain that responsibility falls on health care providers for instrumental communication, whereas it falls on patients for the negotiation of values? Four elements may contribute to our understanding of the attribution of responsibility: the legal framework, the training of health professionals, the organisational culture, and the adaptation to communication as a prerequisite to other adaptation issues.

Firstly, the Belgian legislation on patient rights [47] states that health professionals have to provide *all information which concerns the patient and may be necessary to understand his health status and its probable evolution*. Moreover, this information must be delivered *in clear language*. This statement clearly places the legal responsibility for communication on the health professional. Moreover, the provision of clear information is needed to obtain the informed consent of the patient. As physicians are mostly in charge of informing patients and obtaining their consent for treatment, it is thus understandable that they are more likely to place greater responsibility on health professionals than are other health professionals such as nurses or allied health professionals. The Belgian law on patient rights, however, does not deal with the negotiation of values between health professionals and patients. In fact, the legislation on patient rights does not deal with patients’ preferences: it mainly focuses on informed consent and the refusal of health care [47]. Moreover, if a patient is not satisfied with the services provided by health professionals, the patient has the right to consult another health professional or to be treated in another health service. Choosing the health professional is an important right in the Belgian health system which, in a sense, may shift the responsibility of adaptation on the patient shoulders [45,47,48].

Secondly, the training curricula for health professionals are still for the most part concerned with the acquisition of knowledge and practical skills. All non-technical skills, such as empathy or kindness, are assumed to be part of the personality of the health professional and are not strongly emphasised in the curriculum. As most health professionals adapt a paternalistic style of communication, they may not perceive the need to negotiate on patients’ values, especially in the Belgian context where patients may change health professionals or health services if they are not satisfied with them [49-53]. It could, as a consequence, be easier for health professionals to place more responsibility for the negotiation of values on patients than on health professionals.

Moreover, in the absence of formal training in cultural competence, Belgian health professionals identified the lack of knowledge and skills in cultural competence as one of their main difficulties when caring for migrants [54]. Supporting and organising formal training in cultural competence for health professionals has been recommended by experts in order to promote fair health care for migrants and ethnic minorities in Belgium [55,56].

Thirdly, the attribution of responsibility may depend on the organisational culture, norms, and beliefs in the health services. Indeed, a health service is more likely to develop culturally competent care if the organisational culture makes resources available to cope with ethnic diversity [12,25,26]. Primary care services may feel less responsible because they count with fewer human resources compared with hospitals. Consequently, when compared to hospitals, primary care services have fewer opportunities to avail of interpreters or support for the provision of information in several languages. Intercultural mediators, funded by the Belgian federal state, are restricted to general and psychiatric hospitals, although some Internet-based mediation initiatives are currently being developed for primary care services [57]. These initiatives, thus, remain less accessible to primary care services or solo health professionals such as general practitioners, physiotherapists, and home nurses.

Fourthly, the adaptation to communication issues may be a prerequisite to other adaptation issues. In other words, when health professionals cannot communicate with migrant patients because of language barriers, they may be less likely to adapt their practices to the needs of migrant patients [54,58]. When language barriers arise, health professionals are less likely to identify the emotions of the patients and more likely to fail to identify the needs of the patients [59,60]. Moreover, health professionals may be more supportive of resources that benefit the health professionals themselves [61]. Interpreters, for example, facilitate diagnosis and the elaboration of a treatment plan. So it makes sense for health professionals to support the implementation of initiatives of that kind, as they are directly related to carrying out their job. They will reduce diagnosis errors, the length of stay in emergency rooms or in general hospitalisation, the number of additional examinations, and, ultimately, will reduce the costs of hospitals and primary care services [62].

Compared to the study by Hudelson and her colleagues [41], our study relied on a more diverse sample of health professionals. While Hudelson and her colleagues only sampled physicians and medical doctors, our sample was made up of nurses, health assistants, physicians, paramedics, and administrative staff in outpatient and inpatient services. However, we did not observe significant differences between professions, even though a previous study reported such differences [63]. One major difference between the results of Hudelson and her colleagues and our study concerns gender concordance. While, in the Hudelson study, professionals declared that they would adapt to a gender concordance request made by a patient, in our study, professionals declared that patients have to adapt to the gender of the health professional. This difference could be partly explained by a statement issued by the National Council of Physicians of Belgium, which repeated, in a recent statement, that the patient could not choose the gender of the health professional in emergency situations [64].

Our study has some limitations. Of the three health services that declined participation, two were considered to be highly exposed to immigration. This could affect our results: health professionals may feel less responsibility for adapting health care to migrants when they are more exposed to migrant patients. Accordingly, our results may represent a “best” case of the Belgian situation. Further studies should involve a different sample, with a view to ensuring a balanced representation of the different levels of exposure to immigration.

The second limitation is related to the first. As the management of cultural and ethnic diversity in health care remains a sensitive topic, we cannot underestimate the risk of a social desirability bias at the individual level. Again, our results may depict a more positive situation than actually exists.

## Conclusions

To our knowledge, this study is one of only a few that have looked at the question of who feels responsible for the adaptation of health care in Europe. To conclude, we would like to highlight two key messages. First, responsibility for adaptation is multidimensional, depending on the nature of the adaptation required by the situation. Health professionals may feel more responsible when the adaptation in question is directly connected to their professional roles, such as having the right information in the right place and at the right time to provide adequate treatment. Negotiation between patients and health professionals remains marginal.

Second, health professionals felt they shared the responsibility with patients. If health professionals do not feel responsible for adaptation, they are less likely to be involved in culturally competent health care. Further studies should investigate the relationship between the responsibility of health professionals and the development of culturally competent interventions. Moreover, further studies could usefully pay more attention to health professionals’ perception of their responsibilities.

## Endnotes

^1^As there is no ethnic census in Belgium, these data are the only official source of statistics on the foreign population in Belgium.

^2^No respondent came from a European country outside the European Union. If any had, we would have added an additional category labelled “European outside EU-27”.

## Abbreviations

COMETH: Competences in ethnicity and health; OECD: Organisation for economic co-operation and development.

## Competing interests

The authors declare that they have no competing interest.

## Authors’ contributions

MD designed the protocol of the study, collected the data, carried out the analysis of the findings of the COMETH project. VL helped to design the protocol, drafted the manuscript, and contributed to the analysis of the findings. Both authors contributed to the drafting of the manuscript and gave their approval to the final version of the manuscript.

## Pre-publication history

The pre-publication history for this paper can be accessed here:

http://www.biomedcentral.com/1472-6963/14/294/prepub
